# Does entrepreneurship education in China develop entrepreneurial intention? the role of self-efficacy and experience

**DOI:** 10.1371/journal.pone.0286090

**Published:** 2023-07-19

**Authors:** Ju Xu, Yitu Fu, Xueying Zhang

**Affiliations:** 1 School of Economics and Management, Shanghai Ocean University, Shanghai, China; 2 School of Information Management, Shanghai Lixin University of Accounting and Finance, Shanghai, China; 3 AIEN Institute, Shanghai Ocean University, Shanghai, China; Wroclaw University of Economics and Business: Uniwersytet Ekonomiczny we Wroclawiu, POLAND

## Abstract

Entrepreneurship education has attracted much attention in recent years. However, the relationship between entrepreneurship education and entrepreneurial intention has not achieved an agreement yet. To reconcile these conflicting conclusions, we explore the effect of entrepreneurship education on entrepreneurial intention from the content of the entrepreneurship education programs and different types of individuals who have participated in the program. Leveraging the self-efficacy theory and event system theory, we examine the mediation of entrepreneurial self-efficacy from five dimensions and the moderation of entrepreneurial experience. The sample of this study comprised 243 individuals who participated in entrepreneurship education in China (female = 40.3%, The majority of responders with an age range from 21 to 30 years). The results reveal that entrepreneurship education has a significantly positive influence on entrepreneurial intention (β = 0.331, p < 0.001). Entrepreneurial self-efficacies in searching (β = 0.382, p<0.001), planning (β = 0.249, p<0.001), and marshaling (β = 0.134, p<0.05) play mediating roles in the relationship between entrepreneurship education and entrepreneurial intention. We also find that entrepreneurial experience negatively moderates the relationship between entrepreneurship education and entrepreneurial intention (β = -0.212, p<0.05). The results have implications for entrepreneurship education scholars and policymakers in China.

## Introduction

Over the last decade, there has been rising research in exploring the factors for sustainable growth in emerging economies, such as structural change factors [1] and education on environmental quality [2]. Entrepreneurship is regarded as a powerful tool for social and economic transformation because it not only promotes economic growth and market innovation but also provides more jobs to improve employment [3]. Encouraging people to create new ventures can promote global efforts to achieve prosperity and eliminate poverty.

As a critical step in promoting entrepreneurship activities, entrepreneurship education programs have attracted the attention of many scholars, which enables people to gain knowledge for future-oriented decisions [4,5]. Especially, with the “massive entrepreneurship and innovation” policy, entrepreneurship education in China is regarded as an essential way to encourage sustainable development and it becomes a hot topic in both entrepreneurship and education research fields.

The effect of entrepreneurship education has attracted the attention of scholars [6–8]. However, existing research on the effect of entrepreneurship education on entrepreneurial intention has not achieved a consistent conclusion yet [8,9]. While several studies suggest that entrepreneurial education has a significantly positive effect on entrepreneurial intention [8,10–12], other studies find no effects on entrepreneurial intention [13,14]. At the extreme, some scholars even believe that the relationship is significantly negative [15]. Perhaps the content of the programs and different types of individuals are most likely to affect outcomes. Therefore, our study attempts to investigate the mechanism of the relationship between entrepreneurship education and entrepreneurial intention to reconcile the contradiction.

Entrepreneurial self-efficacy (ESE) has been widely recognized as a critical cognitive factor in the field of entrepreneurship research, and its predictive effect on entrepreneurial intention and venture performance has attracted more attention [16–18]. But at present, the majority of research on entrepreneurial self-efficacy in entrepreneurship education field is in the form of single-dimension measurement. It not only limits the depth of exploration of entrepreneurial self-efficacy but also is not conducive to guiding the implementation of specific content of entrepreneurship education in practice. McGee et al. (2009) suggests entrepreneurial self-efficacy is a multi-dimensional construct and this multi-dimensional construct widely used in recent studies [16,18]. This task-specific entrepreneurial self-efficacy is divided into five phases, namely searching for new business ideas, planning marketing and financing strategies, marshaling, implementing with people, and implementing with finance, which is coherent with the basic content of entrepreneurship education. Therefore, in this paper, we use this multi-dimension construct to explore the roles of different dimensions of entrepreneurial self-efficacy in the relationship between entrepreneurship education and entrepreneurial intention. It helps to find out the impact of the content of the programs.

Different types of individuals may have different learning outcomes [8]. Previous studies have explored several individual factors that influence entrepreneurial intention, such as gender [19], dark personality traits such as Machiavellianism, narcissism, and psychopathy [20], and outcome expectations [21]. However, the impact of previous entrepreneurial experience has not been fully studied yet. Individuals with prior exposure to entrepreneurship may have higher perceptions of entrepreneurial desirability and feasibility [22], and benefit less at the margin because they already have high levels of resources and capabilities before the program [8]. For individuals without previous entrepreneurial experience, entrepreneurship education programs are relatively novel to these people, and they may further cause a change in an individual’s cognition and behavior [23]. In this paper, we leverage event system theory to examine whether the effectiveness of entrepreneurship education varies with prior entrepreneurial experience.

We do the research using China as our research context. This is a unique context for studying the effectiveness of entrepreneurship education. As an emerging economy, China has embraced the importance of entrepreneurship education and made it a national priority to develop entrepreneurship in 2014 [24]. However, entrepreneurship education in China is still young. Its teaching pedagogies have not formed an integrated system yet. There are some issues of contradiction on the teachers, teaching content, and students [25]. Until now, there are only a few pieces of literature introducing entrepreneurship education phenomenon in China and it is under-researched [26]. Given these realities, we assert this setting offers an opportunity to examine the relationship between entrepreneurship education and entrepreneurial intention.

We investigate our research questions with a survey dataset. 243 individuals who have participated in entrepreneurship education programs are invited to answer the questionnaires. We use AMOS and SPSS software to analyze the data. We offer three main contributions in this paper. First, we examine how different dimensions of entrepreneurial self-efficacies mediate the effect of entrepreneurship education on entrepreneurial intention. Even though self-efficacy is a growing research issue in entrepreneurship literature [16,21,27], and its meditation role has been the subject of interest in prior research [28,29], our study moves the literature forward by exploring the effect of entrepreneurial self-efficacies in searching, planning, marshaling, implementing with people and implementing with finance phrases on entrepreneurial intention. These task-centered dimensions help to better understand which content of entrepreneurship education is most likely to be impactful on entrepreneurial intention.

Second, we leverage event system theory to introduce the entrepreneurial experience as a moderator. Our results suggest that individuals without entrepreneurial experience are most likely to have higher entrepreneurial intentions after taking entrepreneurship education. We establish the boundaries of the effect of entrepreneurship education. Hence, our study helps understand for whom entrepreneurship education is most likely to be impactful on entrepreneurial intention. Overall, our study reconciles the contradiction we mentioned before [8,9] from the content and the types of individuals’ perspectives.

Finally, our study contributes to the growing stream of research focusing on the effect of entrepreneurial variables in non-Western societies, particularly China [30,31]. We believe that considering the rapid development of entrepreneurship in the Chinese region, understanding the role of entrepreneurship education in this region is crucial.

Therefore, the study aimed to answer the following research questions.

Research question 1: Can entrepreneurship education in China improve entrepreneurial intention?

Research question 2: How do entrepreneurial experience and different dimensions of entrepreneurial self-efficacies affect the relationship between entrepreneurship education and entrepreneurial intention?

**Fig 1:**
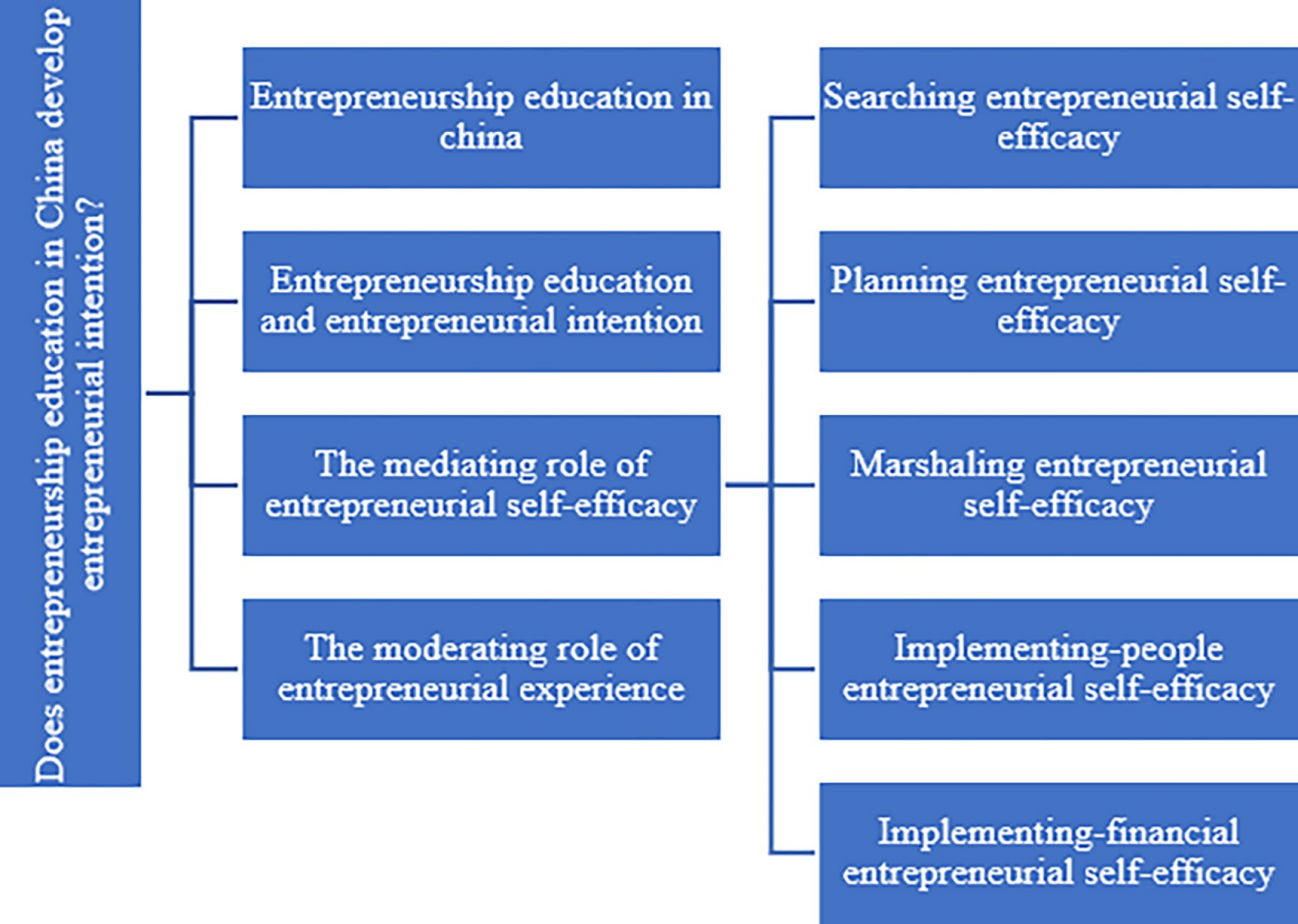
The framework of literature review.

We present our literature review in Fig 1. After introducing entrepreneurship education in China, we first reviewed the relationship between entrepreneurship education and entrepreneurial intention. Then we explore the mediating role of entrepreneurial self-efficacy in the relationship. Specifically, we examined the mediating effects of different dimensions of self-efficacy such as searching, panning, marshaling, implementing with people, and implementing with finance. Finally, we review the moderating role of entrepreneurial experience.

## Theory and hypotheses

### Entrepreneurship education in China

Entrepreneurship education refers to education for entrepreneurial attitudes and skills [9]. Entrepreneurship education in China begins in the 2000s. Under the demand of rapid economic growth, the Ministry of education decided to first launch a pilot stage at nine leading universities across China, such as Renmin University of China, Tsinghua University, and Nanjing University [32]. It aims at improving the students’ entrepreneurial ability and competence, instead of just providing professional skills in traditional education [32]. Since the policy of “massive innovation and entrepreneurship” was put forward in 2014, entrepreneurship education has attracted a great deal of attention in China. It is very common to find an entrepreneurship curriculum in a university [24]. The data shows that 70.4% of colleges and universities hosted entrepreneurial activities, 66.7% of colleges and universities set up entrepreneurial clubs, and 59.7% of colleges and universities established entrepreneurship practice bases. Overall, students in China obtain entrepreneurial knowledge and enjoy the entrepreneurial atmosphere from several aspects, such as lectures and exams given by the course instructor, preparing business plans, pitch competitions [24], meeting with guest speakers with actual entrepreneurial exposure, and business visits and field trips like other countries [33].

However, entrepreneurship education in China is relatively young [26]. There are main issues of entrepreneurship education contradiction, such as structure of teachers being somewhat irrational, teaching content is not comprehensive, and students having weak innovative ideas [25]. It has not yet developed a universally-recognized teaching model with best practices. Many universities and colleges in China cultivate their entrepreneurial education programs in their special ways. For example, Tongji University has established the College of Innovation and Entrepreneurship. Students studying in this college are from different disciplines. Frequent communication among students can promote the transformation of interdisciplinary scientific and technological achievements. The college also provides related policy releases and financing services. Experts inside and outside the college are hired to conduct guidance and regularly organize exchange meetings. Students are encouraged to participate in the "Dream Cup" entrepreneurship competition to practice their entrepreneurial knowledge. Nanjing Forestry University integrates entrepreneurship education into traditional course teaching. The instructors adopt case teaching methods and also organize students to visit enterprises. Harbin Engineering University has established the College of Entrepreneurship Education by establishing a curriculum teaching system, practical training system, and business incubation system. The college provides students with services and guidance in innovation and entrepreneurship competitions and set up workshops to promote students’ entrepreneurial practice activities. Above all, the methods for teaching entrepreneurship in universities and colleges in China varied extensively.

In addition to entrepreneurship education programs provided by state-owned universities, entrepreneurship training institutions run by individuals also provide related entrepreneurship training. For example, several training institutions in Shanghai provide entrepreneurship-related courses. Shanghai Entrepreneurship Education Training Center provides relevant entrepreneurship services. Therefore, individuals in China can obtain entrepreneurial knowledge from different sources.

### Entrepreneurship education and entrepreneurial intention

The intention is usually regarded as the best predictor of planned behavior, especially behaviors which are not easily observed [34]. Planned behaviors can be predicted by the intention to engage in a specific behavior [35]. Entrepreneurial activities are representative of planned behaviors. Entrepreneurial intention is usually defined as one’s desire to start one’s own business [36]. Individuals with high entrepreneurial intentions may finally decide to create a new venture [37]. The determinants of entrepreneurial intention have attracted much attention of researchers. Researchers began to investigate the role of demographic variables. For example, gender is regarded as an important determinant. Entrepreneurs are perceived to have predominantly masculine characteristics, males have higher entrepreneurial intentions than females [10,38]. Entrepreneurial family background, which refers to those people whose close family members are involved in entrepreneurship, will influence the children’s entrepreneurial career choices [9]. Students with an entrepreneurial family background have higher entrepreneurial intentions than those without such a background [39,40]. Personality factors, such as risk propensity [41] and innovativeness [42] play important roles in the emergence of entrepreneurs. Recent studies argue that entrepreneurs are not born to be entrepreneurs, and individuals can be entrepreneurs through learning [43]. Entrepreneurship education is an activity that improves students’ entrepreneurial knowledge and skills, and then finally enhances entrepreneurial intention [44].

However, existing research on the effect of entrepreneurship education on entrepreneurial intention has not achieved a consistent finding [8,9]. Many researchers suggest that entrepreneurial education has a significantly positive impact on entrepreneurial intention [8,10–12], while some studies find no effects on entrepreneurial intention [13,14]. Other studies have also shown that the relationship is significantly negative [15]. To conciliate the contradiction, some scholars believe that the specific nature of entrepreneur courses may lead to different conclusions. For example, Piperopoulos and Dimov find that the relationship between self-efficacy beliefs and intention is negative in theoretically oriented courses and positive in practically oriented courses [45]. Franke and Lüthje (2004) argue that entrepreneurship education enhances entrepreneurial intention in the aspects of initiation, development, and active support [46]. The initiation of entrepreneurship education creates a creative atmosphere to develop ideas for new businesses, stimulates the entrepreneur’s psychological emotions and motivations, and then further enhances an individual’s entrepreneurial intention. In terms of the development of entrepreneurship education, entrepreneurship courses provide students with knowledge and skills [47]. Integration and accumulation of new knowledge are conducive to opportunity recognition and then enhance the perception of opportunities to promote the emergence of entrepreneurial behavior [48]. In terms of active support, entrepreneurship education may provide technical and financial resources. Perceived resource availability is an important factor of perceived feasibility, which in turn promotes entrepreneurial intention [36]. Based on the majority of research on entrepreneurship education and entrepreneurial intention, as well as the context in China, we hypothesize that:

H1: Entrepreneurship education has a significantly positive impact on entrepreneurial intention.

### The mediating role of entrepreneurial self-efficacy

Self-efficacy was first proposed by Bandura (1977) [49]. He believed that self-efficacy is an individual’s self-assessment and judgment for the completion of a certain behavior. Self-efficacy was then introduced into entrepreneurship field. Entrepreneurial self-efficacy (ESE) refers to the belief that individuals believe they can successfully perform various entrepreneurial roles and accomplish various entrepreneurial tasks [50–52]. Simply stated, individuals with high self-efficacy for a certain entrepreneurial task are more likely to persist in that task than those individuals who possess low entrepreneurial self-efficacy.

Numerous studies have validated that ESE is significantly positive to entrepreneurial intention [10,29,51,52]. However, recent studies also find that the relationship between ESE and entrepreneurial intention may be weaker or even nonexistent under some circumstances [18,53]. The general measure of ESE may be the reason for the inconsistent conclusion. Most of the research focuses on the general measure of ESE, which somehow impedes further development and effective application of the construct [54]. In other words, a general measure of ESE fails to provide insight into what specific areas of ESE are most influential to EI.

In this paper, we argue that task-specific types of ESE may influence the ESE-EI relationship. Self-efficacy as first conceptualized by [55] is a task-specific construct, and it is best assessed concerning specific tasks and behaviors. The more task-specific the measure of ESE, the better predictive role efficacy will play in task-specific outcomes [16]. According to the types of tasks [56], identified ESE as opportunity-identification self-efficacy, relationship self-efficacy, managerial self-efficacy, and tolerance self-efficacy, and further explore their individual and unequal impact on entrepreneurial intention and nascent behavior. Mueller and Goic (2003) [57] adopted a four-phase venture creation process model originally proposed by Stevenson, Roberts, and Grousbeck (1985) [58], and constructed a separate measure of entrepreneurial self-efficacy for specific tasks associated with each of the four phases of the process, namely searching, planning, marshaling, and implementing. They empirically confirmed the construct’s multi-dimensional nature and also reported that an individual’s level of entrepreneurial self-efficacy varied by phases. Based on the former study, McGee(2009) divided the implementing phase into implementing with people and implementing with finance phase [54]. The searching phase refers to opportunity identification. The planning phase refers to converting opportunities into a visible business plan. The marshaling phase refers to assembling resources to create a new business. The implementation phase refers to managing business related to people and finance. ESE in these phrases means individuals have confidence in these tasks that come later in the new venture creation process such as planning, marshaling of resources, implementing, and then creating a new venture [54].

Entrepreneurship education can improve ESE [10,45,59,60]. We argue that the EE-ESE relationship can apply to ESE in specific phrases. In the Planning phase, ideas and actions will be converted into a viable business plan. Saeed (2015) pointed out that, in addition to providing entrepreneurial knowledge and skills needed as the traditional role of entrepreneurship education, entrepreneurial awareness, motivation, and ideas will be inspired in this early starting phase, to further develop entrepreneurial self-efficacy [59]. In the searching phase, entrepreneurs will form ideas and discover unique opportunities. Entrepreneurship education helps entrepreneurs with related knowledge, to develop new products and provide specific services, and learn the opportunity produced in entrepreneurship [61]; individuals who received entrepreneurship education will balance linear and nonlinear thinking modes and form thinking modes with the use of data based on evidence [62]. It helps improve creative thinking ability, enhances the ability to explore and identify business opportunities, and finally improves entrepreneurial self-efficacy [63]. In the marshaling phase, entrepreneurs will have access to capital, labor, consumers, and suppliers to realize ideas. Entrepreneurship education not only provides individuals with experience and knowledge, but also significantly improves risk assessment and identification, resource utilization, value creation, and relational network skills, and also provides a supportive environment for individual entrepreneurship [64]. In the implementing phase related with people, an excellent entrepreneur should deal with the relationship with supply customers, employees, and providers of capital. The growth of enterprises requires entrepreneurs to have the ability to solve problems efficiently and quickly. Human capital in the field of entrepreneurship needs to provide specific products and services and solve consumer problems. Entrepreneurship education needs to promote the formation of human capital in this field [61]. Concerning the implementing phase related with finance, entrepreneurs are the main risk bearers of new ventures with financial pressure for the long-term growth and success of enterprises. Kraaijenbrink believes that entrepreneurship education provides individuals with social network resources in specific technical areas, such as financial management and one-to-one support [65].

The mediating role of ESE in entrepreneurship literature is widely explored [29,66–68]. We found the link between EE and ESE. Meanwhile, ESE is related to entrepreneurial intention. Thus, we put forward the following hypotheses:

H2a: Searching entrepreneurial self-efficacy mediates the relationship between entrepreneurship education and entrepreneurial intention.
H2b: Planning entrepreneurial self-efficacy mediates the relationship between entrepreneurship education and entrepreneurial intention.
H2c: Marshaling entrepreneurial self-efficacy mediates the relationship between entrepreneurship education and entrepreneurial intention.
H2d: Implementing-people entrepreneurial self-efficacy mediates the relationship between entrepreneurship education and entrepreneurial intention.
H2e: Implementing-finance entrepreneurial self-efficacy mediates the relationship between entrepreneurship education and entrepreneurial intention.

### The moderating role of entrepreneurial experience

While several studies suggest that EE has a significantly positive effect on entrepreneurial intention [8,10–12], other studies find no effects on entrepreneurial intention [13,14] or even significantly negative [15]. Maybe moderators have not been examined systematically across studies [69]. In this paper, we argue that the relationship between entrepreneurship education and entrepreneurial intention is contingent on whether the individuals have entrepreneurial experience.

Entrepreneur experience refers to the knowledge and skills that entrepreneurs acquired in their previous experiences related to entrepreneurship, including rational and perceptual concepts [70]. Entrepreneur experience helps to improve entrepreneurial intention. For example, the process of using entrepreneurial experience to overcome obstacles increases the possibility of exploiting entrepreneurial opportunities and enhancing entrepreneurial intention [71]. Entrepreneurs’ ability to leverage their business ownership experience is associated with their product and work practices innovation [72]. Entrepreneurial experience has a positive effect on entrepreneurial intention, even if it is a failure experience. Business failure may bring financial and emotional costs to entrepreneurs [73]. When people face losses, they will enhance their entrepreneurial self-efficacy [29], seek risk-taking behaviors to compensate for the losses, and simulate their intention to continue their business.

Event system theory suggests that the strength of the events influences individuals and organizations [23]. Novelty is one of the strengths of events, which means the extent to which an event is different from the present and previous behaviors and events. Novel events will break the routine and trigger in-depth interpretation and a change of behavior, because existing rules or procedures are not likely applied to novel events [74]. For individuals who do not have entrepreneurial experience, the content learned in EE is brand new and with a high novelty. It leads individuals to change their original awareness and generate active analysis. For individuals who already have entrepreneurial experience, the content learned in EE may be involved in the previous entrepreneurial process. Therefore, the novelty of entrepreneurial education is relatively lower, which will not significantly improve the individuals’ intention to start a business. Therefore, we propose that the EE-EI relationship is weakened by entrepreneurial experience.

Hypothesis 3: Entrepreneurial experience negatively moderates the positive relationship between entrepreneurship education and entrepreneurial intention.

**Fig 2:**
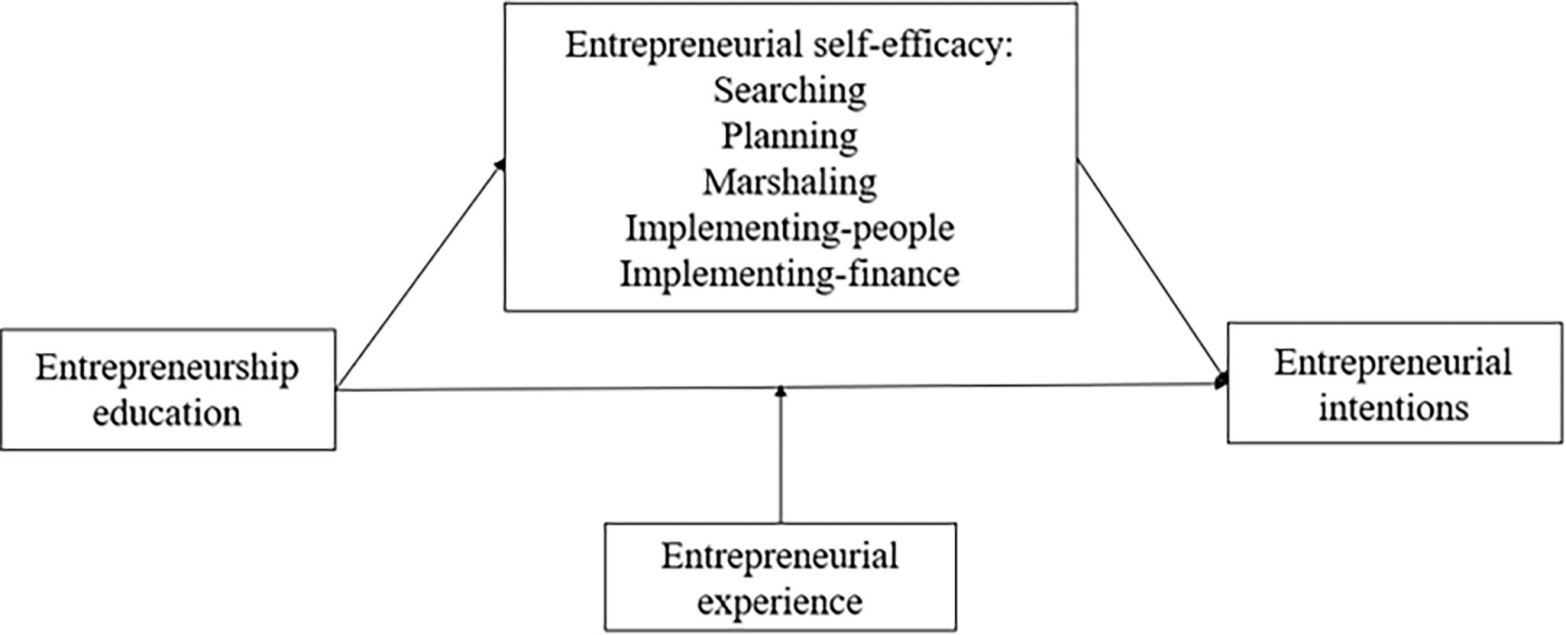
Theoretical model.

We present our theoretical model in Fig 2.

## Methodology

China as an emerging economy country was selected for the present study. Most research on entrepreneurship education has been conducted in advanced countries, with comparatively less research conducted in China. To date, China has established public education systems in universities all over China, which motivates this research. Entrepreneurship education in China is a useful tool for developing entrepreneurial intention among students and is a good context for this research.

Entrepreneurship education, Entrepreneurial self-efficacy, and entrepreneurial experience among potential entrepreneurs could play a vital role in developing newly created ventures in China. Entrepreneurship education is the key determinant of entrepreneurial intention among all these important factors. Our study has societal benefits in terms of evaluating entrepreneurship education. First, policymakers can firmly carry out the benefits of entrepreneurship education, thus responding to the call for a “mass innovation and entrepreneurship” policy in China. Second, educators could focus their attention to provide students with the necessary entrepreneurial knowledge and skills and carry out the most influential activities to improve students’ entrepreneurial intention. Third, Educators may improve their confidence to carry out entrepreneurship education programs with our study results.

### Ethics statement

This study was conducted under the research permit SHOU 21/200329 approved on January 15, 2021, by the Ethics committee of Shanghai Ocean University in Shanghai City in the People’s Republic of China. Interviewing respondents were all above 18 years old. All the respondents of the survey were informed about the study’s aims, voluntary participation, and the right to withdraw at any time. Verbal informed consent was obtained from all participants for inclusion in the study.

### Research method

A questionnaire survey was used to collect data in this research. This research design is pervasive for generating valid and reliable results in research on entrepreneurship education and entrepreneurial intention [27,75]. We adopted the questionnaire as an instrument to collect quantitative data from representatives of the individuals who have received entrepreneurship education.

### Instrument development

The questionnaire contains 36 items. The 6 items related to entrepreneurial intention were adopted from the work of Linan and Chen’s six-item scale [76], and the 19 items related to EES (searching, planning, marshaling, implementation with people, and implementation with finance) were adopted and modified from the work of McGee [54] in our study, and the 6 items related to entrepreneurship education were adopted and modified from the work Franke and Lüthje [44]. A five-point Likert scale is used for most of the items except entrepreneurial experience. Since the survey was carried out to individuals who were Chinese, we followed Brislin’s back-translation procedure [77]. The questionnaire was initially formulated in English, then translated into Chinese by a researcher who is a native Chinese, and then translated back into English by another researcher to see whether the questionnaire was translated accurately. To ensure that our questionnaire was properly understood and completed reliably, we first asked whether the participants had understood the instructions and questions in a pilot survey. we conduct a pilot study with 50 participants who had similar characteristics to those of the final sample. The respondents were asked to highlight the misunderstandings and errors in the questionnaire. We then made the corresponding corrections and modifications, ensuring that all items in the questionnaire can be understood in the right way. With a full understanding of these questions, a large-scale survey is carried out.

### Variables and measures

We tried to adopt mature scales to measure the variables to ensure content reliability. Five-point Likert scales were adopted in our survey ranging from 1(strongly disagree) to 5(strongly agree).

#### Entrepreneurial intention

Entrepreneurial intention was measured by using Linan and Chen’s six-item scale [76] on a five-point Likert scale(1 = strongly disagree, 5 = strongly agree). This scale was widely used to measure entrepreneurial intention. Respondents are asked to respond to the questions, such as “I am ready to do anything to be an entrepreneur”, and “I have the firm intention to start a firm someday”. The item scores were averaged for statistical analyses.

#### Entrepreneurial self-efficacy

Considering entrepreneurship courses in China are generally following specific tasks of managing a small start-up business, we use the measurement of entrepreneurial self-efficacy scale developed by McGee [54] in our study. It includes five dimensions, namely searching, planning, marshaling, implementation with people and implementation with finance, corresponding to five entrepreneurial self-efficacies. The scale consists of 19 items to measure entrepreneurial self-efficacy. The items are on a five-point Likert scale (1 = strongly disagree, 5 = strongly agree).

#### Entrepreneurship education

To explore the mechanism of entrepreneurship education in a more in-depth and accurate way, and based on the actual entrepreneurship education activities, we mainly referred to the scales designed by Franke and Lüthje [46]. They measure entrepreneurship education from the initiation, development, and active support of a total of six items, such as “the creative atmosphere inspires me to develop ideas for new businesses”; “The courses foster the social and leadership skills needed by entrepreneurs”. The items are on a five-point Likert scale (1 = strongly disagree, 5 = strongly agree). In the tables following behind, it is listed shortly as “EE”.

#### Entrepreneurial experience

Entrepreneurial experience was measured as a dummy variable by the following question “Have you ever had entrepreneurial experience before you joined the entrepreneurship education project?” 0 represents the respondent does not have any entrepreneurial experience and 1 represents the respondent has entrepreneurial experience.

#### Control variables

According to previous studies, we control for demographic variables related to entrepreneurial intention, including gender, age, entrepreneurial family background, and education. Gender is a dummy variable, coded as 0 for males and 1 for females. Entrepreneurial family background is a dummy variable. 0 represents the respondent who doesn’t have an entrepreneurial family background and 1 represents the respondent who has an entrepreneurial family background.

#### Data collection

**Fig 3:**
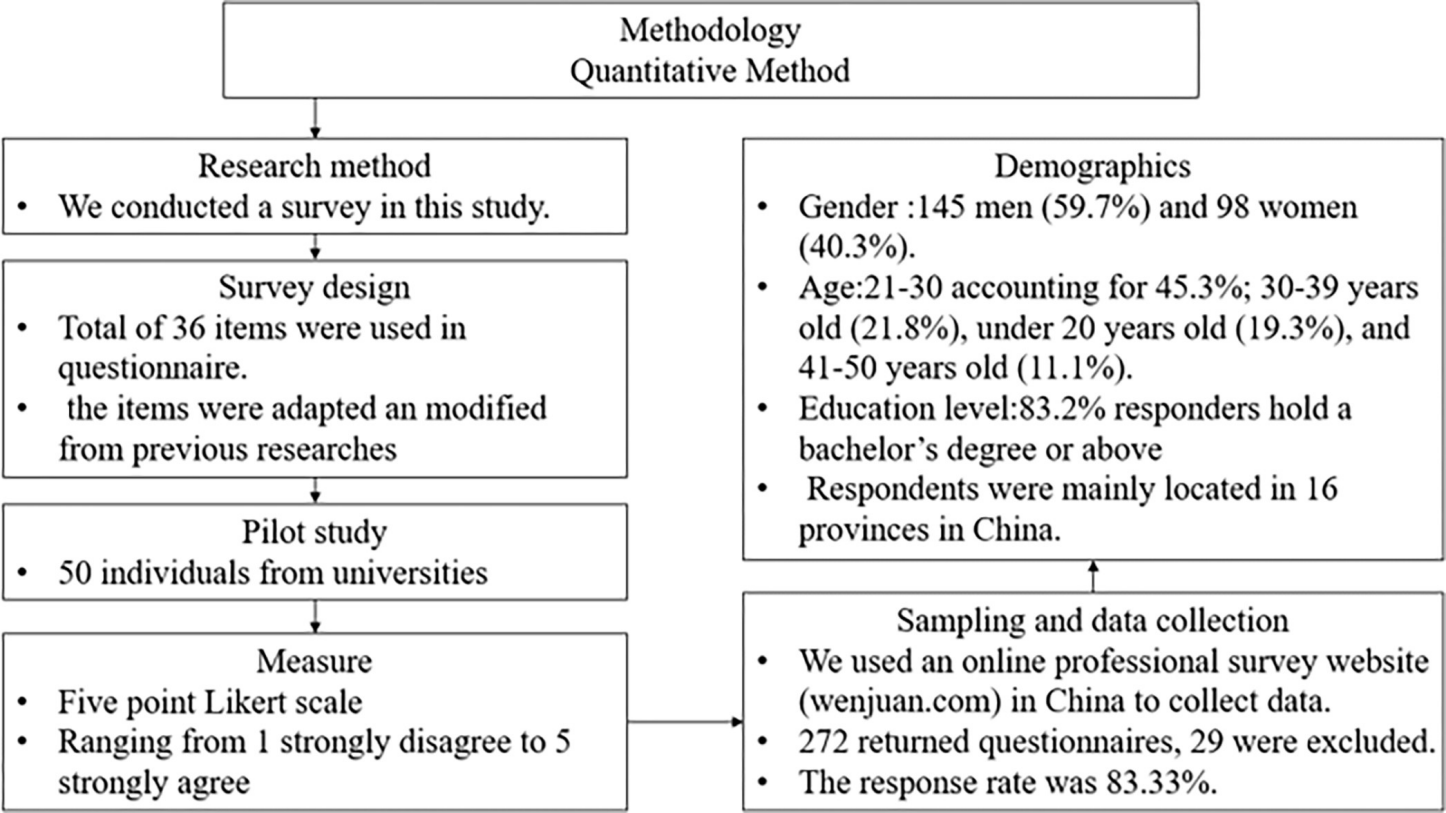
Methodology chart.

We collect data from universities and organizations in China. The samples were composed of individuals who participated in entrepreneurship education programs. We used an online professional survey website (wenjuan.com) in China to collect data. Each participant received a survey link, along with instructions about how to complete the survey. To identify the response of entrepreneurs, we used a screening item asking responders about their occupations. Response is chosen only when it indicated “I have joined an entrepreneurship education program before”. Of the 272 returned questionnaires that have joined the entrepreneurship education programs, 29 were excluded for the following reasons. First, we exclude observations with missing values on variables. Second, we exclude the samples with consistent values from the beginning to the end. After screening out the unusable samples, we had final 243 samples. The response rate was 83.33%. We compared the final sample with the non-used sample using t-tests on gender, age, and education. There is no significant difference between the two groups. Fig 3 shows the methodology chart of our research.

### Demographics

We use SPSS to analyze data. Table 1 provides the demographic characteristics of the sample. The responders were 145 men (59.7%) and 98 women (40.3%). The majority of responders were at their age between 21–30 accounting for 45.3%, followed by 30–39 years old (21.8%), under 20 years old (19.3%), and 41–50 years old (11.1%). 83.2% of responders hold a bachelor’s degree or above. Respondents were mainly located in 16 provinces including the first-tier economic city Shanghai, the Yangtze River Delta Area, and Central and Northeast China. According to the report of Global Entrepreneurship Monitor, there are highly active, generally active, not active, and silent entrepreneurial areas in China. Our sample covered a wide geographical scope and can better represent the actual situation of entrepreneurial activities in China.

**Table 1:** Demographic characteristics of respondents.

Characteristics	Category	Frequency(N)	Frequency (%)
Gender	Male	145	59.7%
	Female	98	40.3%
Age	18–20	47	19.3%
	21–30	110	45.3%
	31–40	53	21.8%
	41–50	27	11.1%
	50+	6	2.5%
Education	High school	21	8.6%
	Bachelor	146	60.1%
	Master	64	26.4%
	Doctor	12	4.9%
Family entrepreneurial background	Yes	211	86.8%
	No	32	13.2%

### Assessing common method bias

The data in our study is from a single survey. There may exist common method variance because of the same data source and the item and measure context [78]. We took the following steps to address the common method concern. First, we conducted a pilot test to check the clearance and accuracy of the items. Second, we promise the anonymity of the respondent in the introduction of the questionnaire, so that respondents may answer the questions according to the real situation. Third, we use different response formats such as a Likert scale and choice questions to reduce the possibility of consistent answers.

We also use statistical methods to verify the common method variance. We carry out exploratory factor analysis with all the items in the questionnaire using Harman’s single factor test. The results of EFA show that all the factors account for 64.524% of the variance. The biggest component accounts for 32.078% of the variance. Therefore, it indicates that the single factor does not exceed 50% of covariance, and does not account for the majority of covariance. There is no serious common method bias in our study.

### Reliability and discriminant validity

To determine the potential dimensions of entrepreneurship education, we use SPSS to conduct exploratory factor analysis. The results show that the KMO statistic value is 0.805, greater than 0.5; The result of Bartlett’s spherical test is less than 0.05, so the test result shows that it is suitable for factor analysis. The absolute values of correlation coefficient between any measurement items are larger than 0.3, so all the items of the scale are retained. The factor is extracted according to the standard that the eigenvalue is greater than 1. The result shows that six items fall on a common factor, and the factor loads are greater than 0.5. The average variance extraction (AVE) is 0.51, which is higher than the minimum acceptable standard of 0.5, indicating that the variable has good validity. Amos was used for confirmatory factor analysis. The fitting index results of single factor model showed that χ 2 / DF = 1.979, less than 3, RMSEA = 0.064, less than 0.08, IFI = 0.986, CFI = 0.985, TLI = 0.963, AGFI = 0.943, all values are greater than 0.9, so the fitting result is good. Cronbach α is 0.784 and the combined reliability is 0.860. It means that it has good internal consistency and good reliability. Therefore, the entrepreneurship education scale has good reliability and validity, and it can be used for subsequent empirical analysis.

We use SPSS21 statistical analysis software to analyze the internal consistency of the measurement scale. The results show that Cronbach’s α values of entrepreneurship education is 0.784, entrepreneurial efficacies are 0.841, 0.769, 0.730,0.819,0.769; entrepreneurial intention is 0.868, respectively. The indices suggest that the reliability of each scale is around 0.7 and is acceptable. The factor loads of variables are greater than 0.5, indicating that the variables have good validity. Table 2 shows reliability and validity of variables in our study.

**Table 2:** Reliability and validity of variables.

Variables	Items	Factor load	AVE	CR	Cronbach’s α
Entrepreneurship education	EE 1	0.569	0.51	0.86	0.784
	EE 2	0.627			
	EE 3	0.736			
	EE 4	0.796			
	EE 5	0.792			
	EE 6	0.732			
Searching entrepreneurial efficacies	SESE 1	0.89	0.761	0.905	0.841
	SESE 2	0.864			
	SESE3	0.862			
Planning entrepreneurial efficacies	PESE 1	0.804	0.593	0.853	0.769
	PESE2	0.737			
	PESE 3	0.777			
	PESE 4	0.761			
Marshaling entrepreneurial self-efficacy	MESE 1	0.619	0.653	0.849	0.730
	MESE 2	0.731			
	MESE 3	0.608			
Implementing-people entrepreneurial self-efficacy	IPESE 1	0.741	0.528	0.869	0.819
	IPESE 2	0.761			
	IPESE3	0.814			
	IPESE 4	0.776			
	IPESE 5	0.646			
	IPESE 6	0.599			
Implementing-finance entrepreneurial self-efficacy	IFESE1	0.839	0.685	0.867	0.769
	IFESE 2	0.844			
	IFESE 3	0.799			
Entrepreneurial intention	EI 1	0.717	0.605	0.901	0.868
	EI 2	0.782			
	EI 3	0.809			
	EI 4	0.829			
	EI 5	0.687			
	EI 6	0.831			

We conducted a confirmatory factor analysis with all the data items to verify the validity of the model. We use Amos 22 to test the model. The results showed that fit indices such as IFI, CFI and TLI values of the model were better than recommended values 0.9; the RMSEA value was better than recommended values 0.08; and the CMIN/DF was better than recommended values 3. The indices suggest that the data have a good fit for the model.

## Results

The research problem of this study is to investigate whether and how entrepreneurship education programs affect entrepreneurial intention. We begin this section by displaying the descriptive statistics and correlations of all variables. We then use SPSS21 statistical software to analyze the data. We first investigate whether entrepreneurship education affects entrepreneurial intention. We then explore the mediating role of five dimensions of entrepreneurial self-efficacy to testify the content of entrepreneurship program matters. Finally, we examine whether there is heterogeneity in the relationship between entrepreneurship program and entrepreneurial intention. Namely, we consider whether the entrepreneurship education differentially impacts entrepreneurial intention based on their skills and knowledge, measured by entrepreneurial experience. Our findings have implications for the literature on entrepreneurship education and entrepreneurial intention.

### Descriptive statistics and correlations

Table 3 displays the descriptive statistics and correlations for all variables in our study. From the correlations in this table, we can see that entrepreneurship education and entrepreneurial experience were positively correlated with entrepreneurial intention. All dimensions of entrepreneurial self-efficacy (searching, planning, marshaling, implementing with people, and implementing with finance) are significantly correlated to entrepreneurial intention. This suggests that it is appropriate to examine the relationships among these variables in the subsequent hypothesis testing.

**Table 3:** Mean, standard deviation (SD), and coefficient of each dimension by Pearson’s correlation analysis.

	Mean value	SD	1	2	3	4	5	6	7	8	9	10	11	12
1. Gender	1.40	0.492	1											
2. Age	2.32	0.990	-0.097	1										
3. Education	3.11	0.936	-0.107	0.175**	1									
4. Entrepreneurial family background	0.87	0.339	-0.077	0.151*	0.033	1								
5. Entrepreneurship education	3.591	0.543	-0.026	0.58**	0.044	0.024	1							
6. SESE	3.554	0.768	-0.124*	0.305**	0.129*	0.244	0.46***	1						
7. PESE	3.4568	0.638	-0.145*	0.241**	0.081	0.198**	0.474***	0.762**	1					
8. MESE	3.768	0.653	-0.089	0.448**	0.159*	0.173**	0.438***	0.607**	0.605***	1				
9. IPESE	3.792	0.671	-0.137*	0.327**	0.089	0.161*	0.576**	0.559**	0.585**	0.671**	1			
10. IFESE	3.787	0.583	-0.153*	0.225**	0.026	0.127*	0.543**	0.485**	0.522**	0.462**	0.623**	1		
11. Entrepreneurial Experience	0.4	0.492	-0.1	0.502**	-.040	0.155*	0.098	0.368**	0.306**	0.35**	0.162*	0.213**	1	
12. Entrepreneurial intention	3.532	0.736	-0.160*	0.359**	-0.047	0.210**	0.379***	0.705**	0.674***	0.589***	0.541***	0.417***	0.455***	1

Notes: n = 243. Standardized coefficients are reported. **p≤0.01, *p≤0.05(two-tailed tests).

Among the control variables, gender has a negative correlation with entrepreneurial intention, which means that men show higher entrepreneurial intentions than women. This is consistent with present research conclusions on the relationship between gender and entrepreneurial intention [10,38].

### Analyses and results

We use SPSS21 statistical software to analyze the data. We followed a step-by-step regression analysis on the structural model to test our hypotheses. Table 4 presents the results of the hypothesis. Model 2 shows that entrepreneurial education has a significant positive impact on entrepreneurial intention (β_1_ = 0.331, p_1_ < 0.001). Hypotheses1 is supported.

**Table 4:** Verification of the mediating effect of entrepreneurial efficacy.

Variables	Entrepreneurial intention			
	Mode 1	Mode 2	Mode 3	Mode 4
Gender	-0.128*	-0.125*	-0.066	-0.064
Age	0.346***	0.346***	0.130**	0.129**
Education	-0.127*	-0.127*	-0.174***	-0.174***
Entrepreneurial family background	0.152*	0.153**	0.019	0.017
Entrepreneurship education		0.331***		-0.015
SESE			0.382***	0.384***
PESE			0.249***	0.250***
MESE			0.134*	0.135*
IPESE			0.087	0.092
IFESE			-0.055	-0.051
R^2^	0.428	0.538	0.609	
ΔR^2^		0.275	0.594	
F			40.4***	

Model 3 presents that the coefficient of searching entrepreneurial self-efficacy, planning entrepreneurial self-efficacy, and marshaling entrepreneurial self-efficacy on entrepreneurial intention are positive and significant (β_2_ = 0.382, β_3_ = 0.249, p<0.001; β_4_ = 0.134, p<0.05 respectively). However, the regression coefficient of implementing-finance entrepreneurial self-efficacy and implementing-people finance entrepreneurial self-efficacy on entrepreneurial intention are not significant. The reason may be that different types or dimensions of entrepreneurial self-efficacies may have individual and different relationships for different dependent variables, especially for entrepreneurial intention and start-ups [54]. Compared with implementing stage, entrepreneurial self-efficacy in searching, planning, and marshaling can stimulate individual entrepreneurial intention, while self-efficacies in implementing with people and finance stage focus on promoting sustained and steady growth of enterprises in the future.

In model 5–9 in Table 5, entrepreneurial education has a significantly positive influence on all dimensions of entrepreneurial self-efficacies with regression coefficients respectively β = 0.420, 0.445, 0.375, 0.536, 0.515. p< 0.001. Finally, in model 4, SESE, PESE, and MESE have significantly positive influences on entrepreneurial intention with respectively regression coefficients 0.384, 0.250, p<0.001; 0.135, p<0.05. Meanwhile, entrepreneurial education has no positive influence on entrepreneurial intention. This means SESE, PESE, and MESE play completely mediate roles in the relationship between entrepreneurial education and entrepreneurial intention. Therefore, H2a, H2b, H2c are supported. H2d and H2e are not supported.

**Table 5:** The relationship between entrepreneurship education and entrepreneurial self-efficacy.

Variables	Entrepreneurial Self-efficacy (ESE)				
	SESE	PESE	MESE	IPESE	IFESE
	Mode 5	Mode 6	Mode 7	Mode 8	Mode 9
Gender	-0.072	-0.106	-0.029	-0.092	-0.122*
Age	0.190**	0.133*	0.357***	0.214***	0.156**
Education	0.062	0.021	0.074	0.014	-0.040
Entrepreneurial family background	0.198***	0.159**	0.105*	0.108*	0.083
Entrepreneurship education	0.420***	0.445***	0.375***	0.536***	0.515***
R^2^	0.317	0.292	0.357	0.410	0.347
ΔR^2^	0.302	0.277	0.343	0.398	0.334
F	21.95***	19.516***	26.267***	32.946***	25.22***

**Fig 4:**
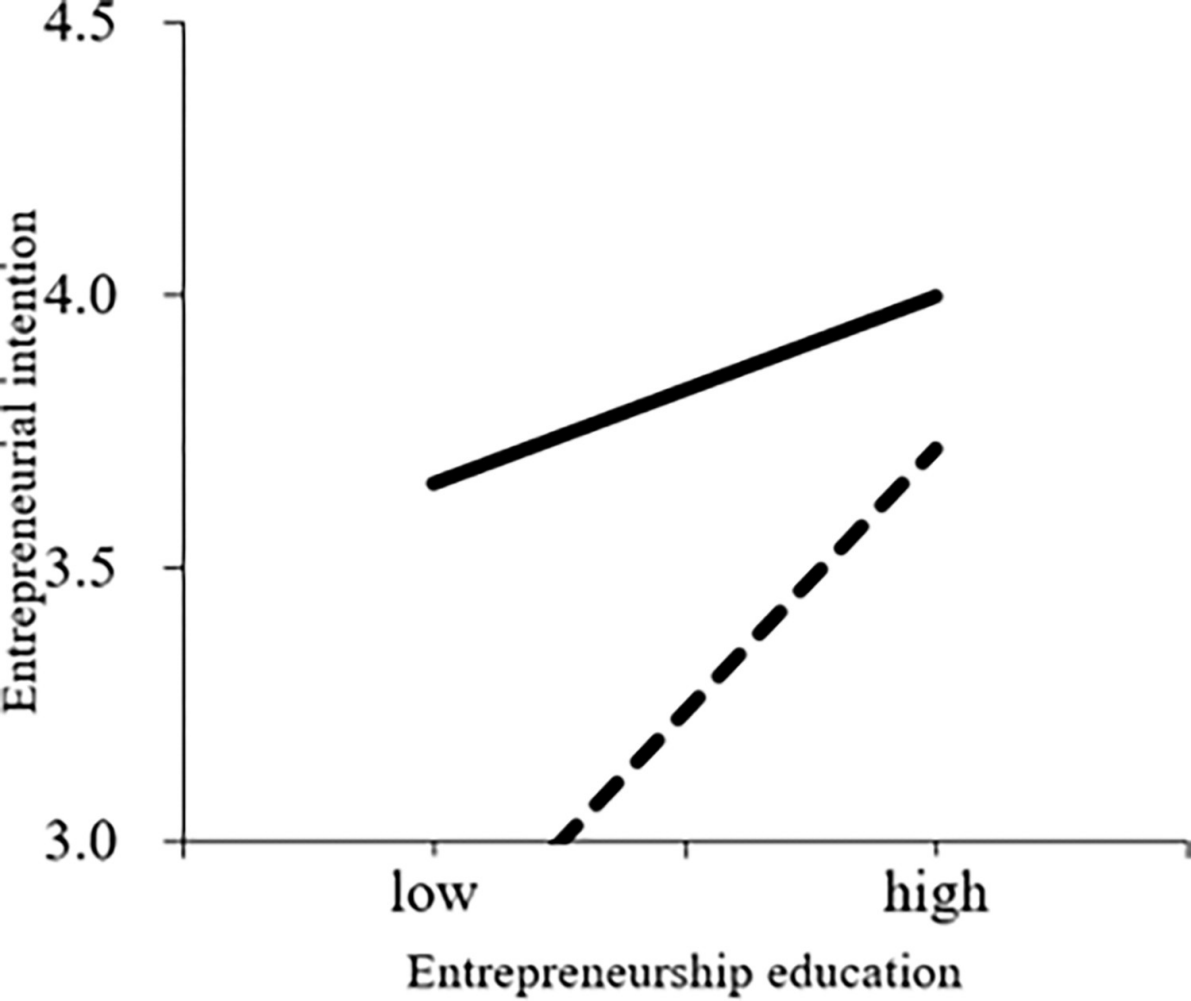
The moderating role of entrepreneurial experience in the relationship between entrepreneurship education and entrepreneurial intention.

In model 11 in Table 6, entrepreneurial experience has a significantly positive influence on entrepreneurial intention with a regression coefficient of 0.375, p<0.001. In model 12, the interaction of entrepreneurship education and entrepreneurial experience is negative to entrepreneurial intention, with the regression coefficient -0.212, p<0.05. H3 is supported. Fig 4 portrays the moderating role of entrepreneurial experience in the relationship between entrepreneurship education and entrepreneurial intention.

**Table 6:** Verification of the moderating effect of entrepreneurial experience.

Variables	Entrepreneurial intention		
	Model 10	Model 11	Model12
Gender	-0.128*	-.091	-0.103*
Age	0.346***	0.137*	0.104
Education	-0.127*	-0.092	-0.092
Entrepreneurial family background	0.152*	0.114*	0.100*
Entrepreneurship education		0.306***	0.443***
Entrepreneurial Experience		0.375***	0.399***
Entrepreneurship education * Entrepreneurial Experience			-0.212**
R^2^	0.183	0.399	0.424
ΔR^2^	0.169	0.383	0.407
F	13.315***	26.061***	24.683***

Note. ***p≤0.001, **p≤0.01,*p≤0.05.

## Discussion

This study aimed to explore the relationship between entrepreneurship education and entrepreneurial intention. Using data from the survey of 243 individuals who received entrepreneurship education, this study provides an analysis of how entrepreneurship education affects the effectiveness of entrepreneurial intention in China.

The results of the study significantly extend the existing studies in several ways. First, the result shows that entrepreneurship education is positively related to entrepreneurial intention in China. Adopting a more rigorous measure of entrepreneurship education, we find that entrepreneurial education positively affects entrepreneurial intention, which conforms with prior research [9,22]. Our result also validates prior findings based on a dummy measure of entrepreneurship education [79]. This responded to the call for the use of a more rigorous measurement of entrepreneurship education [80]. On the other hand, this finding also contributes to the research on the effect of entrepreneurship education and entrepreneurial intention in the Chinese Context. Like entrepreneurship education programs in the U.S. [29], Latin American [81] and Chile [82], entrepreneurship education in China has a positive effect on entrepreneurial intention. Our result is also a complement to research on the outcome of entrepreneurship education in the Chinese context [83,84].

Second, the role of entrepreneurial self-efficacy in the relationship between entrepreneurship education and entrepreneurial intention was identified. The results indicate that entrepreneurial self-efficacies at searching, planning, and marshaling phrases play mediating roles between entrepreneurship education and entrepreneurial intention, while implementing with people and finance entrepreneurial self-efficacies don’t have such mediating roles. We offer a richer understanding of the mediating role of entrepreneurial self-efficacy scholars have discussed by Zhao (2005). He found that the effects of perceived learning from entrepreneurship-related courses, and risk propensity on entrepreneurial intention were fully mediated by entrepreneurial self-efficacy [29]. Our results show that different dimensions of entrepreneurial self-efficacy have different mediating roles between entrepreneurship education and entrepreneurial intention. The results also enrich the multi-dimensional construct of ESE research proposed by McGee et al. (2009). McGee et al. (2009) divided ESE into five phases, namely searching for new business ideas, planning marketing and financing strategies, marshaling, implementing with people, and implementing with finance. This multi-dimensional construct widely used in recent studies [14,16]. Our research extends this line of research by exploring the different effects of multi-dimensional ESE on entrepreneurial intention.

Third, the role of entrepreneurial experience in the relationship between entrepreneurship education and entrepreneurial intention was explored. We find that entrepreneurial experience is positively related to entrepreneurial intention. The result is consistent with previous studies [22,85,86]. Prior entrepreneurial experience facilitates one’s learning of practical knowledge about the specific processes of entrepreneurship and then improves the entrepreneurial intention. We further find that entrepreneurial experience negatively moderates the influence of entrepreneurship education on entrepreneurial intention. The complementary effect of entrepreneurial experience to that of entrepreneurship education is proposed and testified. The result is consistent with previous studies. Zhang found that the mediating effects of entrepreneurial learning on entrepreneurial intention via attitudes and perceived behavioral control respectively is moderated by exposure to entrepreneurship [22]. Our finding adds nuance to previous contradictory studies of the effect of entrepreneurial education on entrepreneurial intention. Individuals with different backgrounds may have different entrepreneurial intentions after they receive entrepreneurship education.

## Theoretical and practical contributions

### Theoretical implications

Our findings have several implications for the literature on entrepreneurship education. First, the previous research on the measurement of entrepreneurship education ignores the differences in the degree of entrepreneurship education received by individuals as it is usually measured as a dummy variable. More rigorous research is needed to measure the impact of entrepreneurship education [87]. To explore the mechanism of entrepreneurship education more deeply and accurately, we break through the previous research model of entrepreneurship education and study the mechanism of entrepreneurship education from individuals’ perceptions. Our study provides a new perspective to research entrepreneurship education.

Second, the relationship between entrepreneurship education and entrepreneurial intention has not reached a consistent conclusion [8,9]. To reconcile these conflicting predictions, we find that the content of the programs and different types of individuals affect the outcomes of entrepreneurship education. We theorize a mediation model and test the boundary condition.

We find that the content of entrepreneurship education matters to the effectiveness of entrepreneurship education. We offer a richer understanding of the mediating role of entrepreneurial self-efficacy scholars have discussed [29]. We find that entrepreneurial self-efficacies at searching, planning, and marshaling phrases play mediating roles in the relationship between entrepreneurship education and entrepreneurial intention, while implementing with people and finance entrepreneurial self-efficacies don’t play mediating roles. In this way, we can see that the content of entrepreneurship education matters to the effectiveness of entrepreneurship education. The EE program provides more skills and knowledge in searching for an opportunity, planning the entrepreneurship process, and marshaling the resources helps to improve individuals’ entrepreneurial intention.

Then, we examine the different types of individuals that affect the outcomes of entrepreneurship education. We set the boundary of the effectiveness of entrepreneurship education on entrepreneurial intention. Our results reveal that the effect of entrepreneurship education on entrepreneurial intention is weaker when individuals have had entrepreneurial experience before. Our findings are consistent with previous findings [88], individuals that already have pre-program entrepreneurial resources and capabilities will benefit less at the margin [8]. Our study contributes to the study of entrepreneurship education by reconciling the contradiction.

Third, we study the effectiveness of entrepreneurship education leveraging the self-efficacy theory and event novelty from event system theory. We explore the role of education and experience on entrepreneurial intention in an integrated framework. Education programs and experience are both learning sources [86,89,90]. Our study empirically verifies the substitution and complementary relationship between education and experience.

### Practical implications

This study also provides practical implications for entrepreneurial education. First, educators and policymakers can firmly carry out the benefits of entrepreneurship education, thus responding to the call for a “mass innovation and entrepreneurship” policy in China. Second, educators should pay attention to the construction of an entrepreneurship education system. In the process of entrepreneurship education, we need to carry out entrepreneurial education activities and form an atmosphere that generates inspiration. Faculties should be well-trained to provide the student with the necessary entrepreneurial knowledge and skills. Meanwhile, a support system with finance and a network could be set up to provide active support. Third, we need to pay attention to the individual’s knowledge structure. Entrepreneurship education is a complementary way of experience to obtain skills and knowledge. Educators may have the confidence to provide entrepreneurship education.

## Limitations

Although we have provided some insights into the previous study, our study still has its limitations. First, we use cross-section data to predict the relationship between entrepreneurship education and entrepreneurial intention because of the obtainability of the data. However, the effect of entrepreneurship education on consequential entrepreneurial intention and behavior is still needed to be studied by longitudinal data to accurately study the effect. Besides, since we use a questionnaire to collect data, the quality of data may be affected by respondents’ social desirability. We conduct several measures to eliminate the limitation. On one hand, all respondents are informed that there is no standard answer when they are filling in the questionnaires, and all the respondents are anonymous. Therefore, respondents can feel free to complete questionnaires and choose the real answers. On the other hand, we conduct steps to address common method concerns. We analyze data statistically and find that there is no serious common method bias in our study.

Second, our study focuses on entrepreneurial intention to examine the effect of entrepreneurship education, not actual entrepreneurial action. Based on the theory of planned behavior [35], entrepreneurial behavior can be significantly predicted by entrepreneurial intention. Like many other studies on entrepreneurship education, we use entrepreneurial intention as the dependent variable to examine the effect of entrepreneurship education. The settings of this study could be extended to include the actual entrepreneurial activities of the students who join in entrepreneurship education in future research.

Third, our study tests the effect of entrepreneurship education on entrepreneurial intention. Entrepreneurial intention is widely used as the outcome of entrepreneurship education. The entrepreneurial-intention literature assumes that all intending entrepreneurs have similar growth aspirations [91]. Entrepreneurial intention construct is currently too broad and can be made more useful by considering what type of new venture the individual intends to start. we could evaluate the effectiveness of entrepreneurship education combined with the specific aspect of entrepreneurial intention in the future.

Fourth, our research focuses on entrepreneurial self-efficacy to explore the mechanism of the relationship between entrepreneurship education and entrepreneurial intention. Entrepreneurial self-efficacy is an important cognitive factor, which attracts much attention from scholars who research individuals’ behaviors. There is an emerging arena of emotional factors that will also promote individuals to generate different behaviors. We could combine the cognition and emotion factors to comprehensively explore the effect of entrepreneurship education on entrepreneurial intention.

### Future research

First, because of the obtainability of the data, we use cross-section data to predict the relationship between entrepreneurship education and entrepreneurial intention. In the future, a longitudinal design study would add more validity to studies on entrepreneurship education and its outcomes.

Second, our study focuses on entrepreneurial intention to examine the effect of entrepreneurship education. Future studies could be extended to include the actual entrepreneurial activities of the students who join in entrepreneurship education in future research.

Third, although we have tested the effect of entrepreneurship education on entrepreneurial intention, future research may study the effect of entrepreneurship education on growth- and independence-oriented intention [91].

Fourth, both cognitive and emotional factors will influence individuals’ behaviors. Future research can research the interaction of positive feelings (i.e., entrepreneurial passion) with cognition that can have potential contributions toward entrepreneurial intention.

## Supporting information

S1 Data (SAV)
